# Efficacy of Nutrilite® All Plant Protein in Postprandial Glycemic Control: A Non-randomized, Controlled, Crossover Study

**DOI:** 10.7759/cureus.111407

**Published:** 2026-06-24

**Authors:** Madhusudan Channappa, Swati Shukla, Aparna Damle, Palaniyamma Durairaj, Shyam Ramakrishnan

**Affiliations:** 1 Research and Development, NRR Hospital, Bengaluru, IND; 2 Research and Development, Amway Global Services India Pvt. Ltd., Bengaluru, IND; 3 Research and Development, Amway Corporation, Michigan, USA

**Keywords:** diabetes, glycemic control, iauc, nutritional intervention, plant protein, postprandial glucose response

## Abstract

Glucose balance is essential for life and health. It's regulated by organs that absorb glucose after eating and by the liver that produces glucose during fasting. This study aimed to investigate the acute effect of Nutrilite® All Plant Protein (APP), administered as a preload in 250 mL spiced buttermilk, on glycemic response to a standardized 75 g oral glucose load in individuals with compromised blood glucose levels. The impact of APP, a plant-based dietary protein formulation, was assessed using a non-randomized, fixed-sequence, controlled crossover design to evaluate its potential as a nutritional intervention to improve glycemic response. Fourteen participants were enrolled in the study. Efficacy analyses were conducted in the complete paired efficacy population, defined as participants with usable paired glucose measurements at both intervention visits and all predefined time points (n = 10). Blood glucose spikes were better regulated with APP preload at almost all time points compared to the reference product (glucose). In this acute oral glucose tolerance test (OGTT)-based setting, participants maintained glucose values within the normoglycemic range for a longer duration after the APP preload condition than after the glucose-only reference condition. In the complete paired efficacy population (n = 10), the average incremental area under the curve (iAUC) for the reference product was 3049.5 ± 1290.45. In contrast, the average iAUC for APP combined with the reference product was 700.5 ± 688.74, representing a 78% reduction. This decrease in iAUC was statistically significant (p < 0.005). The significant reduction in iAUC suggests that APP may blunt acute glycemic excursions after a 75 g oral glucose challenge. These findings should not be directly extrapolated to mixed-meal settings without additional vehicle-controlled and meal-based studies. Furthermore, the findings should be interpreted as an acute reshaping of the OGTT glucose response curve rather than as evidence of uniformly improved glycemic control throughout the full 120-minute period.

## Introduction

Glucose homeostasis refers to the hormonal and neural regulatory mechanisms that maintain blood glucose levels within a very narrow range [1]. In healthy individuals, the body regulates glucose production and release to ensure sufficient glucose availability to meet the body's demands. In recent years, the rising prevalence of compromised blood glucose levels, including conditions such as impaired glucose tolerance and insulin resistance among the healthy population, has become a focal point in global health research [2,3]. This surge is attributed to a confluence of factors, including sedentary lifestyles, diets rich in refined carbohydrates, and genetic predispositions [4,5]. Plant-based proteins have been shown to positively influence blood glucose control and insulin sensitivity, thereby helping maintain normoglycemia (normal blood glucose levels). Increasing plant-based protein intake is associated with improved metabolic health, including better blood glucose control [6,7].

Blood glucose levels naturally fluctuate throughout the day; however, hypoglycemia, also called low blood glucose, occurs when blood glucose drops below 70 mg/dL [8]. The normal range for blood glucose concentration (glycemia) is 70-140 mg/dL [9]. Hyperglycemia is a condition in which blood glucose exceeds 125 mg/dL fasting and 180 mg/dL two hours postprandially [8]. An individual who has impaired glucose tolerance, or pre-diabetes, has a fasting plasma glucose of 100 mg/dL to 125 mg/dL [8]. The state of normoglycemia is maintained by the complex interactions among hormones that exert hyperglycemic or hypoglycemic effects through alterations in the metabolic pathways that produce or consume glucose [9]. Insulin, produced by beta cells in the pancreatic islets, is the most important hormone for maintaining glucose homeostasis. Insulin secretion is precisely regulated by glucose [10]. Blood glucose levels rise to nearly 140 mg/dL (7.8 mmol/l), or slightly higher, in normal humans after a full meal [8]. Postprandial glycemic response refers to the transient change in blood glucose after a carbohydrate-containing meal or a standardized glucose challenge. In nutrition research, the magnitude, timing, and area under the postprandial glucose curve are used to characterize metabolic responsiveness and food-specific effects. Large interindividual differences in post-meal glucose patterns have been observed even when standardized meals are consumed, supporting the role of meal composition, food matrix, timing, and individual physiology in shaping glucose-response profiles [10,11].

Dietary protein can influence the postprandial glucose profile when consumed with or before carbohydrate. Amino acids may stimulate insulin and incretin responses, while protein preloads may also influence gastric emptying and subsequent glucose appearance. Studies in healthy adults and young adults have shown that protein co-ingestion or premeal protein strategies can reduce or flatten glucose excursions compared with carbohydrate alone [12-16]. The interplay between protein intake, particularly leucine, and glucose intake influences insulin signaling pathways, thereby improving glycemic control. This interaction helps maintain stable glucose levels and improves insulin sensitivity during periods of energy restriction, emphasizing the crucial role of protein in optimizing metabolic outcomes [17]. Several studies have demonstrated that co-ingestion of protein with carbohydrates reduces the glycemic response compared with carbohydrate ingestion alone [18,19]. Similarly, another study reported that co-ingestion of plant protein with rice reduces blood glucose [20]. These findings highlight the importance of considering protein intake as part of a comprehensive dietary strategy for managing blood glucose levels, especially in individuals with compromised blood glucose levels. Hence, this study aimed to evaluate whether APP, administered as a preload in 250 mL of spiced buttermilk 10 minutes before a standardized 75 g oral glucose load, could blunt acute postprandial glycemic excursions, as assessed by incremental area under the curve (iAUC), in individuals with compromised blood glucose levels. Because the comparator was an oral glucose tolerance test (OGTT) rather than a mixed meal, the study was designed as a mechanistic assessment of the acute glycemic response. It was not intended to establish effects in typical meal contexts. The impact of Nutrilite® All Plant Protein (APP) was thoroughly assessed using a non-randomized, controlled, crossover design to provide valuable insights into its potential as a nutritional intervention to improve glycemic response in this specific population. Secondary objectives included safety assessments, adverse event evaluations, vital sign monitoring, and laboratory safety parameter assessments.

## Materials and methods

This study was conducted in compliance with the approved protocol, requirements of the Indian Council of Medical Research (ICMR) ethical guidelines, the International Council for Harmonization of Technical Requirements for Pharmaceuticals for Human Use (ICH) (Step 5) "Guidance on Good Clinical Practice" (E6 R2), Schedule Y, and the Declaration of Helsinki, and any deviations from the requirements have been documented. The study protocol underwent a thorough review and received approval from the ethics committee. It was duly registered with the Clinical Trial Registry India (CTRI) (2024/01/061780) prior to its initiation. The study was conducted from 30 January 2024 to 10 February 2024. The Institutional Ethics Committee of Ashraya Medinova Pvt. Ltd. issued approval ASHIEC-AP-02/2024.

All ingredients used in the study were selected from those permitted under Indian food regulations by the Food Safety and Standards Authority of India (FSSAI). The test product was APP. APP is a neutral-tasting plant protein powder composed of soy protein isolate (82.26%), wheat protein (10%), and pea protein (7.5%). In the test condition, APP was administered as three provided scoops, equivalent to approximately 30 g of powder. This 30 g powder dose provided approximately 24 g of protein, 120 kcal, 0.9 g of carbohydrate, 0 g of total sugars, and 0 g of added sugars. The reference product was 75 g glucose monohydrate. The composition of the spiced buttermilk used in the study is as follows: 100 ml of spiced buttermilk contributed 19 kcal of energy, 1.3 g of total carbohydrate, 1.1 g of protein, and 1 g of total fat. The buttermilk vehicle was used only in the APP preload condition and was not administered during the glucose-only reference condition.

Study design and participant recruitment

This study employed a non-randomized, controlled, crossover design to evaluate the effectiveness of APP in modulating postprandial glycemic response. All participants followed the same fixed sequence: the reference condition was administered first at Visit 2/Day 1, followed by the APP preload condition at Visit 3/Day 4. Thus, the study represents a fixed-sequence within-participant comparison rather than a randomized crossover trial. The same participants served as their own controls, which reduced between-participant variability; however, the absence of randomization meant that treatment effects could not be fully separated from potential order or period effects. Participants were screened and recruited during the screening visit based on eligibility criteria. Written informed consent forms were obtained from all study participants before any study-related procedures were performed. A member of the study team briefed the participants in detail on all study-related procedures.

Each participant who expressed willingness to participate in the study was required to provide the investigator with a fully executed informed consent document before any study-related procedures were undertaken. The investigator retained the original signed document while providing a photocopy to the participant. In obtaining and documenting these informed consents, the investigator meticulously adhered to all pertinent regulatory mandates and strictly followed the principles of Good Clinical Practice. The study population was enrolled as per the participant selection criteria. The study was designed to achieve 90% power, a 95% confidence level, and a 5% significance level. Hence, the sample size was set at 12 evaluable participants, allowing for a 20% dropout rate, for a total of 14 participants in the study.

Participant disposition and analysis populations were predefined for transparent reporting. The enrolled population included all participants who provided informed consent and were enrolled in the study (n = 14). The safety population included all participants who received at least one study intervention and had post-intervention safety assessment data. The complete paired efficacy population included participants who completed both intervention visits and had usable paired blood glucose measurements at all predefined time points for the glucose-only and APP preload conditions (n = 10). Participants with missing post-dose samples due to loss to follow-up were excluded from paired efficacy analyses. No missing glucose values were imputed.

Selection of the participants was based on the following parameters listed. Apparently healthy, normotensive adults aged between 30 and 50 years who met the American Diabetes Association (ADA) 2023 guidelines were enrolled, with fasting glucose levels ranging from 100 to 125 mg/dL. HbA1c levels within the range of 5.7% to 6.4%. Additionally, participants had stable body mass, a BMI ≤30 kg/m², and no history of participation in other clinical trials within the past three months. The exclusion criteria were being on medication or hormone replacement therapy, or undergoing treatment with injectable therapies, including exogenous insulin, glucagon-like peptide-1 (GLP-1) receptor agonists, sodium-glucose transporter 2 inhibitors, or metformin. Participants with allergies or intolerance to soy or wheat were excluded. Participants diagnosed with endocrine hypothalamic-pituitary diseases, women with polycystic ovary syndrome, adrenal disorders, or hypothyroidism, and those of reproductive age who were unwilling to utilize standard contraceptive measures were not considered. Participants with either type 1 or type 2 diabetes or other medical conditions such as chronic kidney disease, stones, liver dysfunction (with elevated enzyme levels), a history of cancer, eating disorders, chronic gastrointestinal disorders (e.g., inflammatory bowel disease or celiac disease), active malignancy, or remission for less than five years after the last treatment and metabolic disorders like hypertension and cardiovascular diseases that might interfere with study outcomes were out of the study.

Participants who had undergone surgical procedures within one month of providing informed consent and were currently participating in, or had previously participated in, a study involving an investigational drug or device were not included in the study. Additionally, participants who were unwilling to comply with study procedures for mental, psychological, or social reasons, or to sign informed consent or adhere to the study protocol, were excluded.

After applying the inclusion/exclusion criteria and accounting for a 20% dropout rate, a total of 14 participants were recruited. Written informed consent forms were obtained from all study participants before any study-related procedures were performed. A member of the study team briefed the participants in detail on all study-related procedures.

Study interventions

At Visit 2/Day 1, all participants received the reference condition first, consisting of 75 g glucose monohydrate administered for the OGTT. No APP or buttermilk vehicle was administered during the reference condition. At Visit 3/Day 4, the same participants crossed over to the test condition, in which APP was administered as 3 scoops (30 g) with 250 mL of spiced buttermilk. A 30 g dose was chosen because it is the maximum recommended daily dose of APP. The APP-buttermilk preload was consumed 10 minutes before administration of the same 75 g glucose monohydrate load [21]. The 10-minute interval was defined as the time between completion of the APP-containing preload and initiation of glucose administration. The APP powder was prepared using the same study product batch and standardized preparation procedure for all participants. Carry-over effects were minimized by the acute nature of the intervention and by the three-day interval between the two intervention visits.

**Figure 1:**
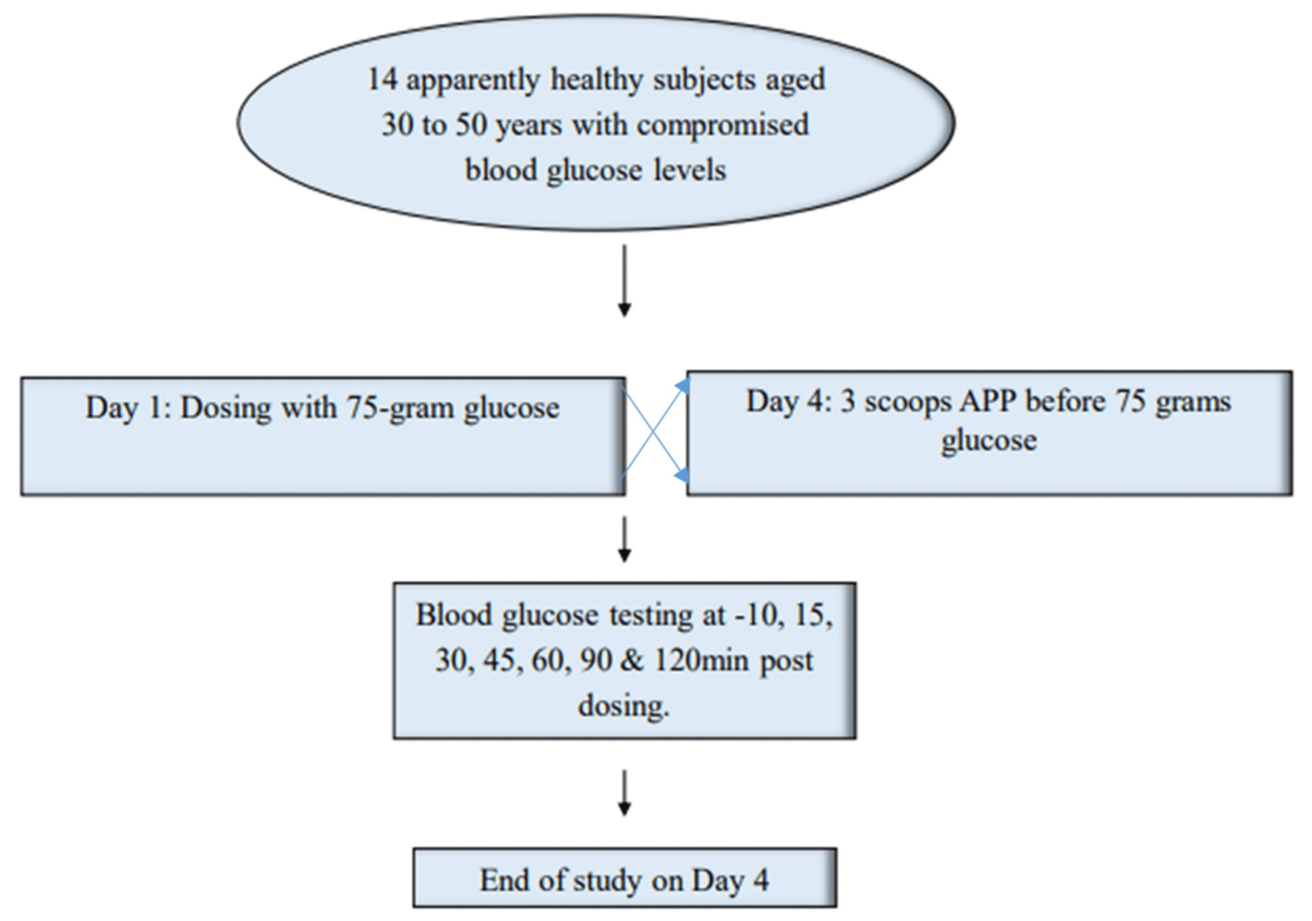
Schematic representation of the fixed-sequence, non-randomized crossover study protocol APP: All Plant Protein

The buttermilk vehicle was standardized across participants with respect to volume, source/preparation, and serving procedure. The reference and test conditions compared a glucose-only OGTT condition with an APP plus spiced-buttermilk preload followed by the same glucose load (Figure 1).

Blood sampling and analysis

The study included multiple visits: screening (Day -7 to 0; Visit 1), baseline (Day 1; Visit 2), and final (Day 4; Visit 3). To minimize between-visit variability, both intervention visits were conducted under standardized conditions. Participants were instructed to attend each intervention visit after an overnight fast of approximately 8-10 hours, maintain their usual dietary pattern between visits, and avoid unusual strenuous physical activity, alcohol, and caffeine for 24 hours before each intervention visit. Visits were scheduled at a comparable time of day. The same glucose dose, APP dose during the test condition, blood sampling schedule, clinical setting, and laboratory procedures were used across intervention visits. The evaluation of postprandial glycemic response was a pivotal component of this study, conducted using a well-defined procedure to assess the impact of APP on it.

During Visit 2/Day 1, participants underwent a 75 g OGTT under the glucose-only reference condition. During Visit 3/Day 4, participants received the APP preload condition, in which APP was administered with 250 mL spiced buttermilk 10 minutes before the 75 g glucose load. For both intervention visits, blood glucose was measured at T0, defined as the sample collected immediately before administration of the 75 g glucose load, and at 15, 30, 45, 60, 90, and 120 minutes after glucose administration. In the APP preload condition, T0 therefore represented the post-preload/pre-glucose value. The -10-minute time point referred to the timing of APP preload administration before glucose dosing and was not used as the baseline for iAUC calculation.

iAUC was calculated separately for each participant and each intervention condition over the 0-120-minute post-glucose window, with T0 as the baseline. Incremental glucose values were calculated as glucose concentration at each post-glucose time point minus the corresponding T0 glucose concentration. Areas above baseline were calculated using the trapezoidal rule. Negative areas below the T0 baseline were not subtracted from the iAUC; when glucose values fell below baseline, the area below baseline was truncated at zero. When a curve crossed the baseline between two adjacent time points, linear interpolation was used to estimate the baseline crossing, and only the area above the baseline was included. The iAUC values are therefore reported as positive incremental glucose exposure (mg·min/dL) from 0 to 120 minutes after glucose administration. Assessments of vital signs and laboratory safety parameters were conducted at screening and at the end of the study visit. Additionally, the assessment of adverse events, if any, was performed at the end of the study.

Statistical considerations

Descriptive statistics including mean, standard deviation (SD), percentage reduction, and change from baseline (CFB) were calculated to characterize glucose responses. The iAUC was computed for each participant using the positive incremental trapezoidal method over the 0-120-minute post-glucose period. T0, defined as the blood glucose value immediately before the 75 g glucose load, was used as the baseline for each condition. Incremental values below T0 were truncated at zero and were not subtracted from total iAUC. Mean iAUC ± SD was then calculated across participants for each condition, and paired t-tests were performed on participant-level iAUC values. Estimated time in glycemic range was assessed as an exploratory descriptive endpoint over the 0-120-minute post-glucose observation period. For each participant and intervention condition, glucose values between consecutive scheduled sampling time points were linearly interpolated. The estimated minutes spent in each predefined glycemic range were summed and divided by 120 minutes to obtain the percentage of observation time in that range. The predefined ranges were hypoglycemia (<70 mg/dL), normoglycemia (70-140 mg/dL), intermediate range (>140-180 mg/dL), and hyperglycemia (>180 mg/dL) and were expressed as mean ± SD. When an interpolated segment crossed a glycemic-range threshold, the crossing time was estimated by linear interpolation. This metric was derived from intermittent OGTT sampling and should not be interpreted as time-in-range from continuous glucose monitoring.

Glycemic response curves were constructed with SD error bars and p-values annotated at each timepoint for CFB comparisons. Paired t-tests were used to compare test and reference products, where the t-value (t = d̄ / SE) indicates the magnitude and direction of the mean paired difference relative to its variability, with larger absolute t-values corresponding to smaller p-values and stronger evidence of a statistically significant difference; p < 0.05 was considered statistically significant. Because all participants received the interventions in the same fixed order, no formal statistical adjustment for sequence, order, or period effects was possible. The paired comparisons should therefore be interpreted as within-participant differences between the Day 1 glucose-only visit and the Day 4 APP preload visit, rather than as treatment effects fully isolated from potential visit-order influences. Missing or unusable glucose measurements were not imputed. Participants with incomplete paired glucose data due to loss to follow-up were excluded from paired efficacy analyses. Therefore, all paired t-tests, iAUC analyses, glycemic response curves, and time-in-range analyses were based on the complete paired efficacy population.

## Results

Participant demographic characteristics

A total of 14 participants were enrolled in the study. Ten participants had complete paired glucose data at both intervention visits and all predefined time points and were included in the complete paired efficacy analyses. Four enrolled participants were excluded from the paired efficacy analysis due to loss to follow-up.

All participants received the investigational products according to the instructions and schedule outlined in the study protocol. A summary of demographic characteristics for the study participants is presented in Table 1. The mean age of the participants was 38.85 years (SD ± 4.91), with a range from 32 to 47 years and a median age of 38 years. The average height was 155 cm (SD ± 3.56), ranging from 150 to 160 cm, with a median height of 155 cm. Participants had an average weight of 57.92 kg (SD ± 9.13), ranging from 41 to 71 kg, and a median weight of 60 kg. The mean BMI was 24.15 kg/m² (SD ± 4.02), with a range from 17.07 to 29.55 kg/m² and a median BMI of 23.73 kg/m².

**Table 1:** Demographic data of enrolled participants Demographic and anthropometric characteristics of study participants at baseline. Data are presented as mean ± SD, range (min, max), and median values for 14 healthy adult participants. SD: standard deviation, min: minimum, max: maximum

	Mean ± SD	Range (Min, Max)	Median
Age (years)	38.85 ± 4.91	32, 47	38
Height (cm)	155 ± 3.56	150, 160	155
Weight (kg)	57.92 ± 9.13	41, 71	60
BMI (kg/m^2^)	24.15 ± 4.02	17.07, 29.55	23.73

Analysis of efficacy outcomes

Mean absolute blood glucose levels are presented in Table 2 to describe the temporal glucose-response profiles under the glucose-only reference condition and APP. Across the 0-120-minute post-glucose period, the glucose-only reference condition demonstrated a spike-and-decline pattern. Mean glucose increased rapidly from T0, 96.4 ± 2.84 mg/dL, to a peak of 157.6 ± 29.5 mg/dL at 45 minutes, followed by a decline to 93.4 ± 22.66 mg/dL at 90 minutes and 84.1 ± 11.37 mg/dL at 120 minutes, which was below the T0 baseline. In contrast, the APP plus spiced-buttermilk preload condition showed a flatter glucose-response profile, with a smaller early rise from T0, 95.3 ± 7.56 mg/dL, to 102.7 ± 11.73 mg/dL at 15 minutes and 107.5 ± 10.5 mg/dL at 30 minutes, followed by values that remained close to baseline during the later phase: 96.5 ± 9.97 mg/dL at 60 minutes, 98.6 ± 8.67 mg/dL at 90 minutes, and 99.1 ± 11.05 mg/dL at 120 minutes. Thus, the APP preload condition appeared to attenuate the early glucose excursion and produce a more sustained glucose profile. In contrast, the glucose-only reference condition showed a rapid rise followed by a sharp decline.

**Table 2:** Mean postprandial glucose levels after the glucose-only reference condition and the APP preload condition Data are presented as mean ± SD (N = 10); statistical analysis was performed using a paired t-test, where the t-value indicates the magnitude and direction of difference between products and the p-value (derived from the t-distribution, df = 9) indicates significance (p < 0.05 = significant, NS = not significant, * = p < 0.05). SD: standard deviation, APP: All Plant Protein

Time point (min)	Reference product (mean ± SD) mg/dL	APP (mean ± SD) mg/dL	% reduction	t-value	Significance of the between-condition analysis (P-value) (paired t-test)
T0	96.4 ± 2.84	95.3 ± 7.56	1.14	0.436	NS
T15	130.3 ± 20.45	102.7 ± 11.73*	21.18	4.8419	0.0009
T30	150.2 ± 25.15	107.5 ± 10.5*	28.43	4.9622	0.0008
T45	157.6 ± 29.5	90.9 ± 17.19*	42.32	6.4155	0.0001
T60	132.1 ± 25.98	96.5 ± 9.97*	26.95	4.3775	0.0018
T90	93.4 ± 22.66	98.6 ± 8.67	-5.57	-0.6847	NS
T120	84.1 ± 11.37	99.1 ± 11.05	-17.84	-3.6965	NS

**Figure 2:**
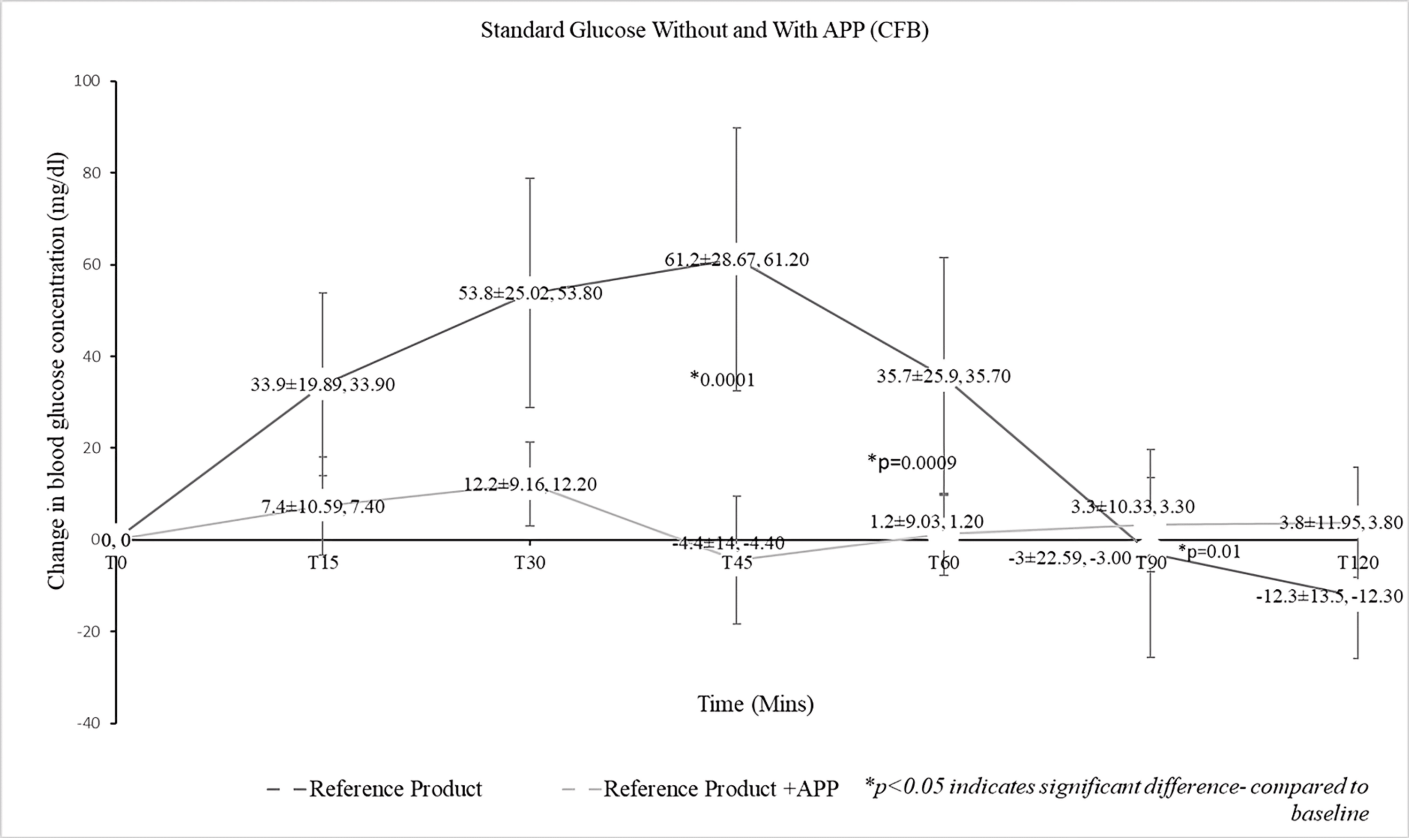
CFB in blood glucose levels after the glucose-only reference condition and the APP plus spiced-buttermilk preload condition Data are presented as mean ± SD (N = 10). Statistical analysis was performed using a paired t-test. A p-value of <0.05 was considered statistically significant. * indicates a significant difference compared to baseline. SD: standard deviation, APP: All Plant Protein, CFB: change from baseline

The CFB values in blood glucose levels for the reference product (glucose) and APP with the reference product at different time points are presented in Figure 2. At baseline (T0), both the reference product and the combination with APP showed no change in blood glucose levels. However, at T15, T30, and T45, the mean change in blood glucose levels for the reference product alone was notably higher than that for the combination with APP (Figure 2). Specifically, at T15, the mean change in blood glucose levels for the reference product (glucose) was 33.9 ± 19.89 mg/dL, whereas for the combination with APP it was 7.4 ± 10.59 mg/dL. Similarly, at T30 and T45, the mean change in blood glucose levels for the reference product (glucose) was higher than that for the combination with APP. The decrease from baseline observed from time point T30 onwards was highly significant (p < 0.05). Notably, at T45, while the reference product (glucose) showed an increase of 61.2 mg/dL ± 28.67, the combination with APP exhibited a decrease of -4.4 mg/dL ± 14. At subsequent time points, a significant reduction in blood glucose levels was observed at T60, whereas at T90 and T120, glucose levels were similar in both groups. The mean change in blood glucose levels for the reference product (glucose) and APP with the reference product at different time points is presented in Figure 2.

Determination of the iAUC

**Figure 3:**
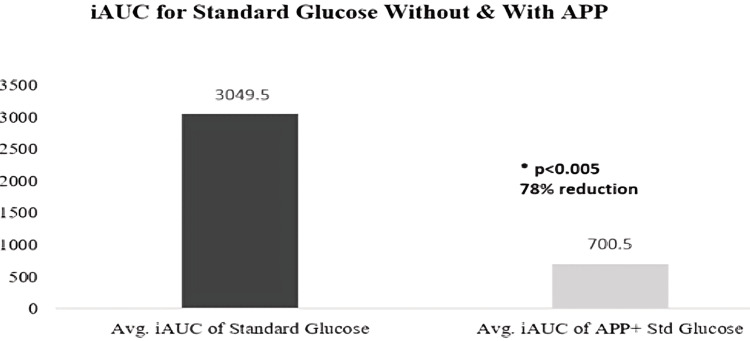
Positive iAUC from 0 to 120 minutes after glucose administration for the glucose-only reference condition and the APP preload condition. iAUC was calculated using T0 as the baseline; negative areas below baseline were truncated at zero and were not subtracted Data are presented as mean values (N = 10). Statistical analysis was performed using a paired t-test. A p-value of <0.005 was considered statistically significant. iAUC: incremental area under the curve, APP: All Plant Protein

Table 3 presents the iAUC values of the study products. iAUC was calculated separately for each participant, with T0 as the baseline immediately before the 75 g glucose load. Areas above baseline were calculated using the trapezoidal rule over 0-120 minutes after glucose administration. Negative areas below baseline were truncated at zero and were not subtracted. Only positive incremental areas above T0 were included; areas below baseline were truncated at zero and were not subtracted. Therefore, the reported iAUC values represent positive post-glucose incremental exposure rather than net AUC. For the reference product, the average iAUC was 3049.5 ± 1290.45. In contrast, when APP was administered in spiced buttermilk 10 minutes before the reference glucose load, the average iAUC decreased significantly to 700.5 ± 688.74, representing a 78% reduction. This decrease in iAUC was statistically significant (p < 0.005) compared with the iAUC of the reference product alone (Table 3, Figure 3).

**Table 3:** iAUC after the glucose-only reference condition and the APP plus spiced-buttermilk preload condition Data are presented as mean ± SD for the complete paired efficacy population (N = 10). Statistical analysis was performed using a paired t-test on participant-level iAUC values. A p-value of <0.05 was considered statistically significant. SD: standard deviation, APP: All Plant Protein, iAUC: incremental area under the curve

Test products	Avg. iAUC (mean ± SD)	% reduction (significance)
Ref. product	3049.5 ± 1290.45	-
Ref. product + APP	700.5 ± 688.74^**^	78% (p < 0.005)

Categories of glucose range and the estimated percentage of observation time in glycemic ranges

**Figure 4:**
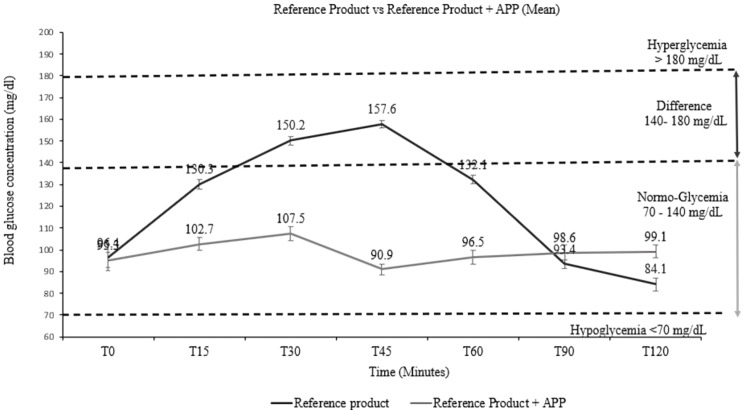
Mean estimated percentage of observation time in glycemic ranges Data are presented as mean ± SD (N = 10). No statistical test was applied for graphical representation unless specified. A p-value of <0.05 was considered statistically significant where applicable. SD: standard deviation, APP: All Plant Protein

The mean estimated percentage of observation time in the different glycemic ranges by the participants is shown in Figure 4. The normal range for blood glucose concentration (glycemia or normoglycemia) is 70-140 mg/dL. Hyperglycemia is a condition in which blood glucose is greater than 125 mg/dL fasting and greater than 180 mg/dL two hours postprandially. At 90 and 120 minutes, blood glucose has risen slightly above baseline but remains within the normoglycemic range on average (Figure 4).

In the exploratory glycemic-range analysis, the APP preload condition was associated with a higher estimated proportion of the 0-120-minute observation period within the normoglycemic range of 70-140 mg/dL compared with the glucose-only reference condition. The estimated normoglycemic-range value was 98.75 ± 18.85% for the APP preload condition and 66.25 ± 23.63% for the glucose-only reference condition. This finding was driven primarily by attenuation of early excursions above 140 mg/dL during the 15-60-minute period (Table 4). These findings suggest that APP administration was associated with a greater estimated percentage of observation time in the normoglycemic range than the glucose-only reference condition. Because the study did not include a mixed meal or a buttermilk-only vehicle control, these results should be interpreted as acute glucose-challenge findings rather than evidence of efficacy across typical meal contexts.

**Table 4:** Comparison of categories in reference and reference product with APP Data are presented as mean ± SD (N = 10). Estimated percentage of observation time in glycemic ranges was calculated as the percentage of total observation time (120 minutes). SD: standard deviation, APP: All Plant Protein

Categories	Range - glucose level (mg/dL)	Standard glucose	APP
		Mean ± SD estimated percentage of observation time in glycemic ranges	Mean ± SD estimated percentage of observation time in glycemic ranges
Hypoglycemia	<70	5 ± 5.95	1.25 ± 1.49
Normoglycemia	70-140	66.25 ± 23.63	98.75 ± 18.85
Range between normoglycemia and hyperglycemia	>140-<180	26.25 ± 14.33	0 ± 0
Hyperglycemia	>180	2.5 ± 1.48	0 ± 0

Safety assessment

All study participants underwent comprehensive clinical examinations, assessment of vital signs, and laboratory tests, suggesting that the investigational product did not adversely affect participants' overall health status and that the study was conducted with a satisfactory safety profile.

## Discussion

Understanding postprandial blood glucose is important not just for those with diabetes or prediabetes; being mindful of this number can help keep one healthy. Long-term high postprandial blood glucose may lead to insulin resistance and poor glucose tolerance [22]. Nutrient preload, by manipulating the sequence of macronutrient ingestion during a meal, is a novel nutritional approach that has proven effective in reducing postprandial hyperglycemia [23]. The beneficial effects of non-carbohydrate protein preloads include promoting insulin and GLP-1 secretion [21].

Postprandial glycemic excursions are relevant not only because they reflect short-term glucose handling, but also because repeated acute glucose peaks may contribute to cardiometabolic risk through oxidative stress, inflammatory activation, and endothelial dysfunction. Human glucose-challenge studies have shown that acute postprandial hyperglycemia can impair endothelial function and increase lipid peroxidation, suggesting a mechanistic link between glycemic variability and vascular stress [24]. In individuals with impaired glucose tolerance, postprandial dysmetabolism has also been associated with oxidative stress, inflammatory markers, and endothelial dysfunction [25]. Therefore, interventions that blunt early postprandial glucose excursions may be relevant to broader strategies to reduce metabolic stress. However, the present study did not directly measure oxidative stress, inflammatory cytokines, endothelial function, insulin, incretins, or gastric emptying.

The outcome of this clinical study provides valuable insights into the efficacy and safety of the investigational product, APP, in managing the postprandial glycemic response. Analysis of efficacy outcomes showed that APP preload was associated with attenuation of the early postprandial glucose rise and a lower overall positive iAUC compared with the glucose-only reference condition. The iAUC decreased markedly when APP was combined with the reference product, resulting in a 78% reduction compared to the reference product alone. These findings suggest that APP preload may blunt the early glycemic excursion after an acute oral glucose load. However, the effect was not characterized by uniformly lower glucose values across all time points, because mean glucose values at 90 and 120 minutes were not lower in the APP preload condition. Our primary finding that pre-meal protein supplementation reduces the prevalence of elevated blood glucose levels corroborates data showing reduced overall hyperglycemia following postprandial glucose-lowering therapies [26].

These effects are consistent with those previously observed after administering a protein preload before an oral glucose load [25,26], supporting this assertion. The present findings can also be interpreted within the broader literature on plant-forward dietary strategies and inflammation pathways. Plant-forward dietary patterns, including Mediterranean-style diets, typically emphasize plant foods such as legumes, fruits, vegetables, whole grains, nuts, seeds, herbs, spices, and sources of unsaturated fat. These patterns provide fiber, polyphenols, antioxidant vitamins, carotenoids, and other bioactive compounds that may influence oxidative stress, inflammatory signaling, endothelial function, gut microbiota, and cardiometabolic health [21-26].

The safety analysis conducted throughout the study period yielded reassuring results. Comprehensive clinical examinations and vital-sign assessments revealed no significant abnormalities or adverse changes among the study participants. Laboratory parameters remained stable, indicating a favorable safety profile for the investigational products. Notably, no adverse events were reported or observed during the study, affirming the safety of APP. The present study was conducted using a standardized 75-g oral glucose load in a controlled clinical setting. This comparator is useful for assessing acute glycemic response under OGTT-based conditions; however, it does not reproduce the physiological complexity of mixed meals, which may contain variable amounts of carbohydrate, fat, protein, fiber, and micronutrients and may produce different gastric emptying, incretin, insulin, and glycemic responses. Therefore, the present findings should be positioned as acute glucose-challenge findings and should not be assumed to apply directly to carbohydrate-rich or mixed-meal contexts without further study.

The reduction in positive iAUC observed in the APP preload condition is consistent with attenuation of the early postprandial glycemic excursion. Several biological mechanisms may contribute to this pattern, including amino acid-stimulated insulin secretion, incretin hormone stimulation, delayed gastric emptying, and altered glucose absorption or disposal [14, 27-29]. However, these mechanisms were not directly tested in the present study because insulin, C-peptide, GLP-1, GIP, glucagon, gastric emptying, oxidative stress biomarkers, inflammatory cytokines, and endothelial function were not measured. Therefore, the present findings should be interpreted as acute OGTT-based glycemic findings, not as direct evidence that APP reduced oxidative stress or inflammation. Future studies should incorporate mechanistic biomarkers to determine whether blunting early glucose excursions with plant-protein preload translates into measurable changes in oxidative stress, inflammation, endothelial function, or other inflammation-related comorbidities.

The exploratory glycemic-range analysis suggested a greater estimated proportion of the 0-120-minute observation period within the normoglycemic range during the APP preload condition. At all time points, APP preload maintained normoglycemic levels, with reduced spikes compared to the glucose-only reference curve, which showed spikes; additionally, there were time points in the range between normoglycemia and hyperglycemia.

The 30 g APP powder dose was selected to provide a physiologically meaningful plant protein bolus of approximately 24 g of protein while remaining within the product's total daily labeled intake. This dose was also selected because protein preload studies have generally used protein amounts within a similar range to influence postprandial glycemic response. Prior studies have reported that whey protein doses of 10-40 g administered before a meal can reduce post-meal blood glucose exposure in a dose-dependent manner, while soy protein isolate preload studies before a 75 g OGTT suggest that larger soy protein doses may be required to produce a clear glucose-lowering effect [14-16,23]. The current study therefore used a practical 24 g protein dose from APP to evaluate whether a plant-protein formulation could attenuate glycemic excursions during an acute standardized glucose challenge.

A 10-minute preload interval was selected as a pragmatic pre-glucose timing intended to allow exposure to the protein preload before the glucose challenge while preserving feasibility for real-world pre-meal use. Previous premeal protein studies have used preload intervals ranging from immediately before meals to 15-30 minutes before carbohydrate or mixed-meal challenges, and recent studies have reported glycemic effects when protein was administered 10 minutes before meals [30,31]. Another important consideration is the administration vehicle. APP was administered with 250 mL spiced buttermilk, whereas the reference condition consisted of glucose monohydrate alone. The contribution of buttermilk in terms of nutritional profile (carbohydrates, protein, and fat) was minimal, although its influence cannot be eliminated. Because the study did not include a buttermilk-only vehicle control, the observed effect cannot be attributed exclusively to APP. The results should therefore be interpreted as the effect of the APP preload as administered in the spiced-buttermilk vehicle.

This study has certain limitations. First, the study used a non-randomized, fixed-sequence crossover design in which all participants received the glucose-only reference condition first and the APP preload condition later. Although the within-participant design reduced between-participant variability and the same glucose dose, sampling schedule, clinical setting, and laboratory procedures were used across visits, the lack of randomization and counterbalanced treatment sequences limits causal inference. Second, the final analysis included a small number of participants and assessed only an acute glucose challenge. The initial sample size (n = 14) was determined in accordance with the methodology described in the referenced study [32]. However, due to loss to follow-up, the final analysis was conducted on 10 participants. To assess the adequacy of this reduced sample size, relevant literature [33,34] was reviewed, which suggests that studies with comparable sample sizes have reported statistically meaningful outcomes, thereby supporting the validity of the present findings.

Third, the study used an acute OGTT-based design with a 75 g oral glucose load rather than a mixed-meal challenge. Although this approach provides a standardized mechanistic assessment of postprandial glycemic response, it limits its generalizability to typical meal contexts, where macronutrient composition, food matrix, fiber, fat content, and meal volume vary. Fourth, APP was administered with 250 mL spiced buttermilk, whereas the reference condition did not include a matched vehicle. Therefore, the independent effect of APP cannot be fully separated from the potential effects of the buttermilk vehicle.

Nonetheless, the intervention effect observed in this study should be interpreted in relation to the specific APP dose and preload timing used. The administered APP dose was 30 g of powder, providing approximately 24 g of plant protein from soy, wheat, and pea. Protein preloads may attenuate postprandial glycemic excursions through several mechanisms, including amino acid-stimulated insulin secretion, enhanced incretin responses such as GLP-1 and GIP, delayed gastric emptying, and changes in insulin clearance. Previous protein preload studies support these mechanisms; however, they were not directly assessed in the present trial. Accordingly, the reduced glucose excursion and iAUC observed after the APP preload condition should be considered an acute glycemic finding, while the underlying mechanisms remain inferential. Future studies should evaluate dose-response effects, compare preload intervals, and include assessments of insulin, C-peptide, GLP-1, GIP, glucagon, and gastric emptying to determine the mechanistic basis of the observed response.

Future studies should include vehicle-matched and energy-matched control conditions, such as glucose alone, glucose plus spiced buttermilk, and glucose plus APP in spiced buttermilk, as well as mixed-meal challenges to determine whether the observed acute effects translate to real-world dietary settings. Finally, the small sample size and non-randomized fixed-sequence crossover design limit the precision and causal interpretation of the findings. Larger randomized, counterbalanced crossover trials or randomized parallel-arm studies with longer follow-up periods, standardized pre-visit dietary and activity monitoring, and broader panels of metabolic biomarkers are warranted to confirm these findings.

## Conclusions

APP, administered as a preload before a 75 g glucose load, was associated with attenuation of glucose spikes and a statistically significant reduction in iAUC compared with the glucose-only reference condition. No adverse events were reported during the study period. The findings suggest that APP preload may help manage acute blood glucose spikes under controlled study conditions.
